# *Helicobacter pylori* Infection Is Associated with Type 2 Diabetes, Not Type 1 Diabetes: An Updated Meta-Analysis

**DOI:** 10.1155/2017/5715403

**Published:** 2017-08-13

**Authors:** Jun-Zhen Li, Jie-Yao Li, Ting-Feng Wu, Ji-Hao Xu, Can-Ze Huang, Di Cheng, Qi-Kui Chen, Tao Yu

**Affiliations:** Department of Gastroenterology, Sun Yat-Sen Memorial Hospital, Sun Yat-Sen University, 107 Yan Jiang Xi Road, 510120 Guangzhou, Guangdong, China

## Abstract

**Background:**

Extragastric manifestations of *Helicobacter pylori* (*H. pylori*) infection have been reported in many diseases. However, there are still controversies about whether *H. pylori* infection is associated with diabetes mellitus (DM). This study was aimed at answering the question.

**Methods:**

A systematic search of the literature from January 1996 to January 2016 was conducted in PubMed, Embase databases, Cochrane Library, Google Scholar, Wanfang Data, China national knowledge database, and SinoMed. Published studies reporting *H. pylori* infection in both DM and non-DM individuals were recruited.

**Results:**

79 studies with 57,397 individuals were included in this meta-analysis. The prevalence of *H. pylori* infection in DM group (54.9%) was significantly higher than that (47.5%) in non-DM group (OR = 1.69, *P* < 0.001). The difference was significant in comparison between type 2 DM group and non-DM group (OR = 2.05), but not in that between type 1 DM group and non-DM group (OR = 1.23, 95% CI: 0.77–1.96, *P* = 0.38).

**Conclusion:**

Our meta-analysis suggested that there is significantly higher prevalence of *H. pylori* infection in DM patients as compared to non-DM individuals. And the difference is associated with type 2 DM but not type 1 DM.

## 1. Introduction

*Helicobacter pylori* (*H. pylori*) is a gram-negative spiral bacterium, colonized in the stomach. Approximately one-half of the population over the world is infected with *H. pylori* [1]. Many researches have proved that *H. pylori* infection is highly associated with gastrointestinal diseases such as chronic gastritis, peptic ulcer disease, gastric cancer, and mucosa-associated lymphoid tissue (MALT) lymphoma since its discovery [2]. In addition, extragastric disorders associated with *H. pylori* infection, such as cardiovascular diseases and metabolic syndrome, have been revealed and some of them were characterized by persistent and low-grade systemic inflammation [3]. Inflammation has been demonstrated to play an important part in the pathogenesis of diabetes mellitus (DM), especially type 2 DM (T2DM) [4]. On the other hand, Kondrashova and Hyöty reviewed that some microbes served as the risk factor participating in the trigger and the development of type 1 DM (T1DM), but some microbes such as *H. pylori* served as a protective factor by lowering the risk of T1DM [5]. Above all, *H. pylori* infection was a factor not negligible in the process of DM.

Since Simon et al. firstly reported the association between *H. pylori* infection and DM [6], many studies were carried out. Several case-control studies have reported a higher prevalence of *H. pylori* infection in DM patients [7, 8]. Some cross-sectional researches also revealed a significant correlation between *H. pylori* infection and diabetes [9–11]. Moreover, a meta-analysis carried out by Zhou et al. suggested a trend toward more frequent *H. pylori* infection in DM patients, especially in T2DM patients [12]. However, Tamura et al. found a significantly higher DM prevalence among individuals with *H. pylori* infection than those without, but the difference could be mostly ascribed to older age [13]. And some studies argued that no difference in the prevalence of *H. pylori* infection was found between DM and non-DM individuals [14, 15]. Overall, this subject remains controversial now.

The present updated meta-analysis was conducted to answer if there is a difference in the prevalence of *H. pylori* infection between DM and non-DM individuals. Subgroup analyses were carried out based on the types of DM, geographical regions, and methods for *H. pylori* detection to further investigate the relationship between *H. pylori* infection and DM.

## 2. Methods

### 2.1. Search Strategy and Selection Criteria

Published guidelines for conducting meta-analyses were followed [16]. We searched PubMed, Embase databases, Cochrane Library, Google Scholar, Wanfang Data (Chinese), China national knowledge database (Chinese), and SinoMed (Chinese) for all relevant articles reported from January 1996 to January 2016, with combinations of the search terms “*Helicobacter pylori*,” or “*H. pylori*,” or “*Campylobacter pylori*,” or “*C. pylori*,” and “diabetes mellitus,” or “diabetes,” or “type 1 diabetes,” or “type 1 diabetes mellitus,” or “type 2 diabetes” or “type 2 diabetes mellitus”.

To be eligible for inclusion, studies had to meet the following criteria: (1) they were published studies which reported *H. pylori* infection in DM individuals and non-DM individuals (individuals without DM, impaired glucose tolerance, or impaired fasting glucose); (2) detailed data of *H. pylori* infection rate in both groups was provided. Studies that did not meet the inclusion criteria were not enrolled.

Studies were excluded if they were as follows: (1) duplicate publications; (2) case report, review, meta-analysis, or guideline; (3) not reporting clinically relevant outcomes; and (4) not providing enough details.

### 2.2. Data Extraction and Quality Assessment

Data were extracted by one investigator, verified by another investigator, and recorded in a well-designed form developed for this study. The data items included authors, year of publication, country, study design, methods of *H. pylori* detection, strains of *H. pylori*, types of DM, age, and sample size. The Newcastle-Ottawa scale (NOS) scoring system was used to assess the quality of the studies [17].

### 2.3. Statistical Analysis

To obtain pooled effect estimates, the random effects model or fixed effects model was used for meta-analysis, according to the heterogeneity among studies. If there was no statistically significant heterogeneity (two-tailed *P* value >0.05) among the pooled studies, the fixed effect model would be applied; otherwise, the random effect model would be applied [18]. Odds ratio (OR) with 95% confidence interval (CI) was used for the case-control and cross-sectional studies, while risk ratio (RR) was for the cohort studies. The presence of between-study heterogeneity was estimated using *Q*-test and *I*^2^ statistics. Sources of heterogeneity were explored by conducting subgroup analyses based on types of DM, geographical regions, and methods of *H. pylori* detection. The two-sided tests with significance level of 0.05 were conducted in pooled analyses and subgroup analyses using RevMan software (Version 5.3 for Windows, Cochrane Collaboration, Oxford, UK). Publication bias was evaluated graphically by the funnel plots and statistically by Begg's test and Egger's test with the STATA software (Version 14.0; STATA Corporation, College Station, TX, US). *Pr* and *P* value less than 0.05 were considered representative of no statistically significant publication bias. If publication bias was indicated, the trim and fill method procedure was performed to identify and correct the publication bias [19]. The basis of the method was to (1) “trim” (remove) the studies causing funnel plot asymmetry, (2) use the trimmed funnel plot to estimate the true “centre” of the funnel, and then (3) replace the removed studies and their missing “counterparts” around the centre (filling). An estimate of the number of missing studies was provided; an adjusted OR is derived by performing a meta-analysis including the filled studies.

## 3. Results

### 3.1. Description of Studies

**Figure 1:**
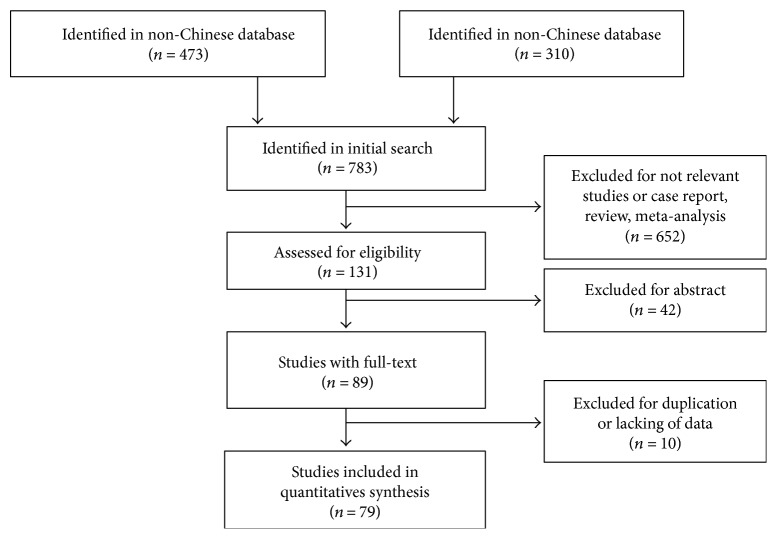
Flow diagram of study selection.

A total of 783 studies were retrieved from PubMed, Embase databases, Cochrane Library, Google Scholar, Wanfang Data (Chinese), China national knowledge database (Chinese), and SinoMed (Chinese). According to the criteria for inclusion and exclusion, 79 studies were included in this meta-analysis (Figure 1). The included study characteristics were summarized in Table 1. All of the articles were qualified to be pooled with quality score of NOS over 5. 76 studies were either case-control or cross-sectional studies, and 3 were prospective cohort ones.

**Figure 2:**
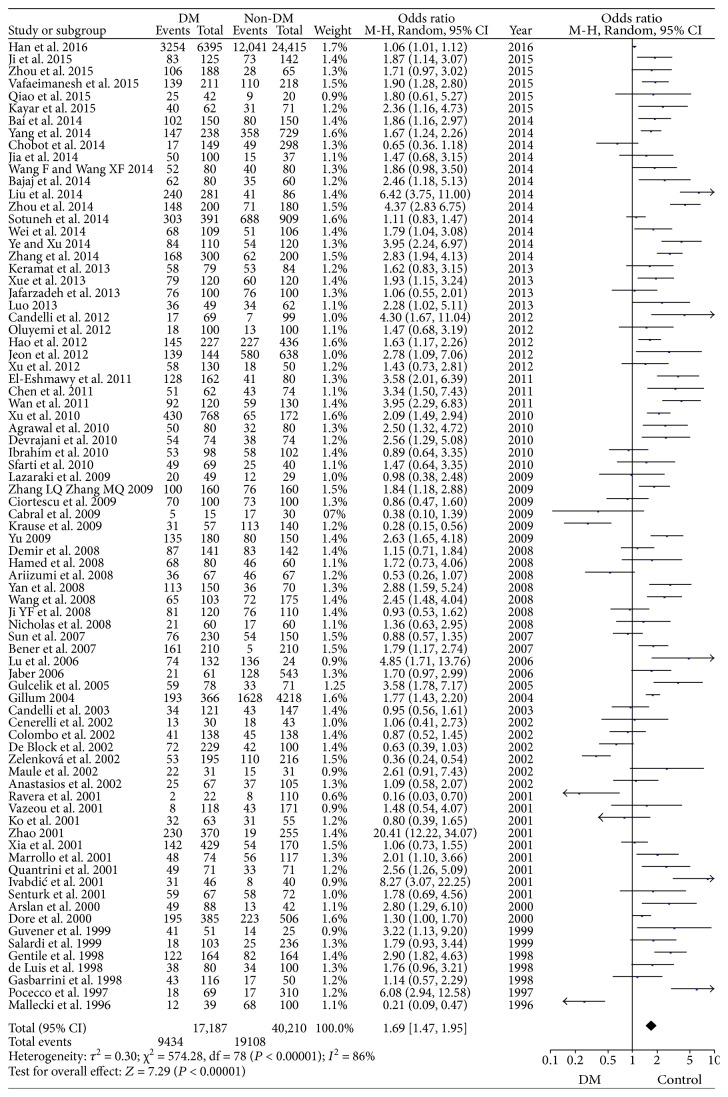
Forest plot for pooled prevalence of *H. pylori* infection in DM group and non-DM group.

A total of 57,397 individuals were enrolled in these studies, with a total *H. pylori* infection prevalence of 49.7% (28,542/57,397). The pooled *H. pylori* infection rate was 54.9% (9434/17,187) in DM group and 47.5% (19,108/40,210) in non-DM group. The OR was 1.69 (95% CI: 1.47–1.95, *P* < 0.001) for the two groups. There was high heterogeneity among the studies (*I*^2^ = 86%). The forest plot for pooled prevalence is showed in Figure 2. Each study was sequentially removed from the analysis, and the adjusted ORs (1.63–1.73) were approximate to the initial ones. Especially, the study of Han et al. [20] recruited a total of 6395 patients in DM group and 24,415 in non-DM group, which accounted for nearly one-third of the enrolled individuals in this analysis. However, after removing the data of Han et al. and re-analyzing, the adjusted odds (OR = 1.71) and heterogeneity (*I*^2^ = 83%) were still approximate to the initial ones in spite of its overweight scale.

### 3.2. Subgroup Analysis

We found a significant association between *H. pylori* infection and DM but the pooled analysis was with high heterogeneity (*I*^2^ = 86%). Subgroup analyses based on the types of DM, geographical regions, and methods for *H. pylori* detection were conducted to detect the sources of heterogeneity.

1. Types of DM

**Figure 3:**
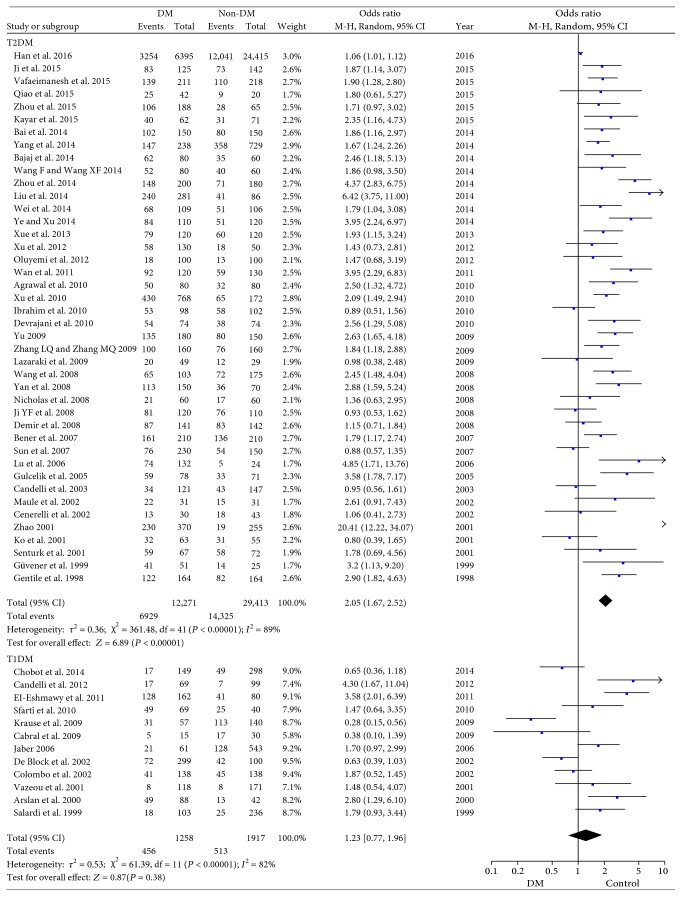
Forest plot for subgroup analysis based on types of DM.

12 studies with 3175 individuals were assigned to the T1DM subgroup, while 42 studies with 41,684 individuals were to the T2DM subgroup. No significant difference was found between T1DM group and non-DM group in *H. pylori* infection rate (OR = 1.23, 95% CI: 0.77–1.96, *P* = 0.38; Figure 3). On the contrary, the pooled data indicated that the prevalence of *H. pylori* infection in T2DM was significantly higher than that in non-DM group (OR = 2.05, 95% CI: 1.67–2.52, *P* < 0.001; Figure 3). Each study including the study by Han et al. with overweight scale was sequentially removed in the subgroups and the adjusted ORs (1.93–2.10 in T2DM and 1.10–1.42 in T1DM) approximated to the initial ones.

(2) Geographical regions

**Figure 4:**
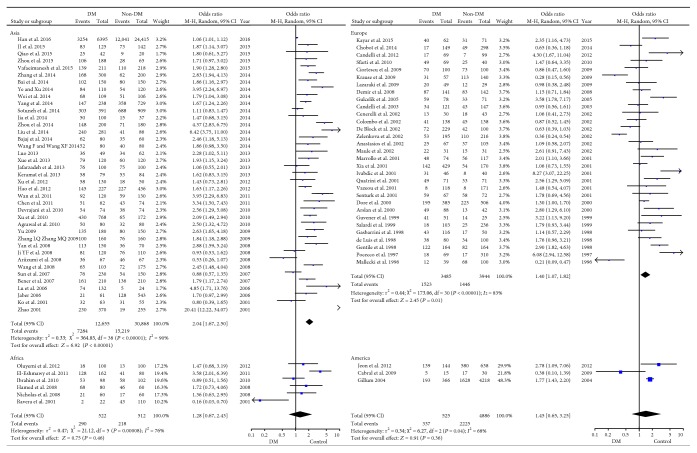
Forest plot for subgroup analysis based on geographic regions. (India, Japan, China, Qatar, Pakistan, Saudi Arabia Iran, Hong Kong, and Taiwan were included in group Asia. Greece, Turkey, Italy, Poland, Romania, Belgium, Spain, Croatia, Israel, UK, and Czech Republic were included in group Europe, as well as Australia because it comprises similar races and people who lived in similar lifestyle with these countries. Brazil and USA were included in group America. Egypt and Nigeria were included in group Africa.)

Subgroup studies stratified by geographical regions were performed. The recruited individuals were mostly from Asia (75.8%, 43,523/57,397). The infection rate was 51.7% (22,503/43,523), 39.7% (2969/7479), 47.3% (2562/5411), and 48.7% (499/1024) in group Asia, group Europe, group America, and group Africa, respectively. No significant difference of *H. pylori* infection rate between DM and non-DM individuals was found in group America and group Africa (*P* = 0.36 for America; *P* = 0.38 for Africa). However, in group Asia and group Europe, significantly higher *H. pylori* infection rate was detected in DM individuals (OR = 2.04 and OR = 1.40, resp.). But there was still high heterogeneity within these subgroups (*I*^2^ = 68%–90%; Figure 4).

(3) Methods for *H. pylori* detection

Methods for *H. pylori* detection displayed different power in accuracy, which consequently might affect the detection rate of *H. pylori* infection. Methods for diagnosis of *H. pylori* were classified as invasive tests and noninvasive tests [21]. Invasive tests included rapid urease test, histology, and culture, and the noninvasive tests included ^13^C or ^14^C urea breath test, stool antigen detection, and serological approaches for antibodies of *H. pylori*. For the serological tests of anti-*H. pylori* IgG or/and IgA antibody in serum, high rates of false-positive results may happen and they cannot identify the differences between the current infection and past infection [21, 22]. So we typically sorted the studies with detection method of serological test into one subgroup and others into the other subgroup as they could identify the current infection precisely.

**Figure 5:**
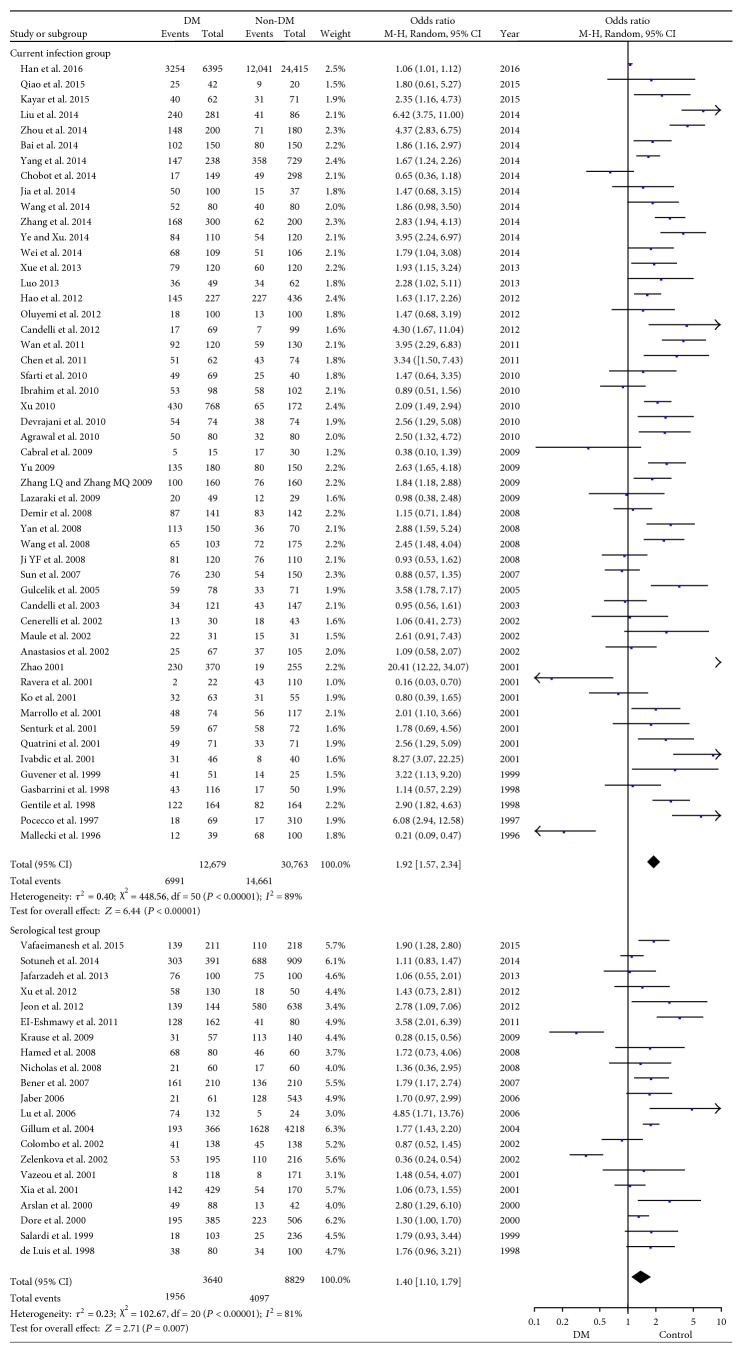
Forest plot for subgroup analysis of methods for *H. pylori* detection.

The studies of current infection group comprised of 51 articles and showed a significant higher prevalence of *H. pylori* infection in DM patients as compared to that in non-DM individuals with OR = 1.92 (95% CI: 1.57–2.34, *P* < 0.001). Similarly, by enrolling 21 articles in serological test group, we found that the infection rate was 53.7% (1956/3640) in DM group while 46.4% (4097/8829) in the non-DM one (OR = 1.40, 95% CI: 1.10–1.79, *P* < 0.001; Figure 5). The heterogeneities in both groups were high among studies with *I*^2^ = 89% and *I*^2^ = 81%, respectively (Figure 5).

### 3.3. Publication Bias

**Figure 6:**
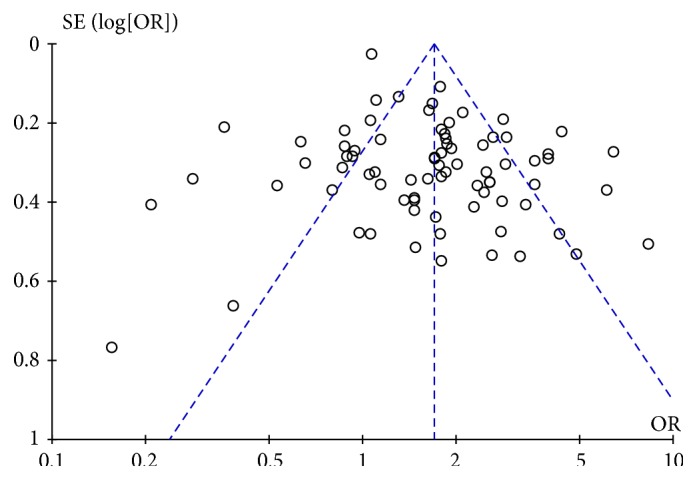
Funnel plot of this meta-analysis.

**Figure 7:**
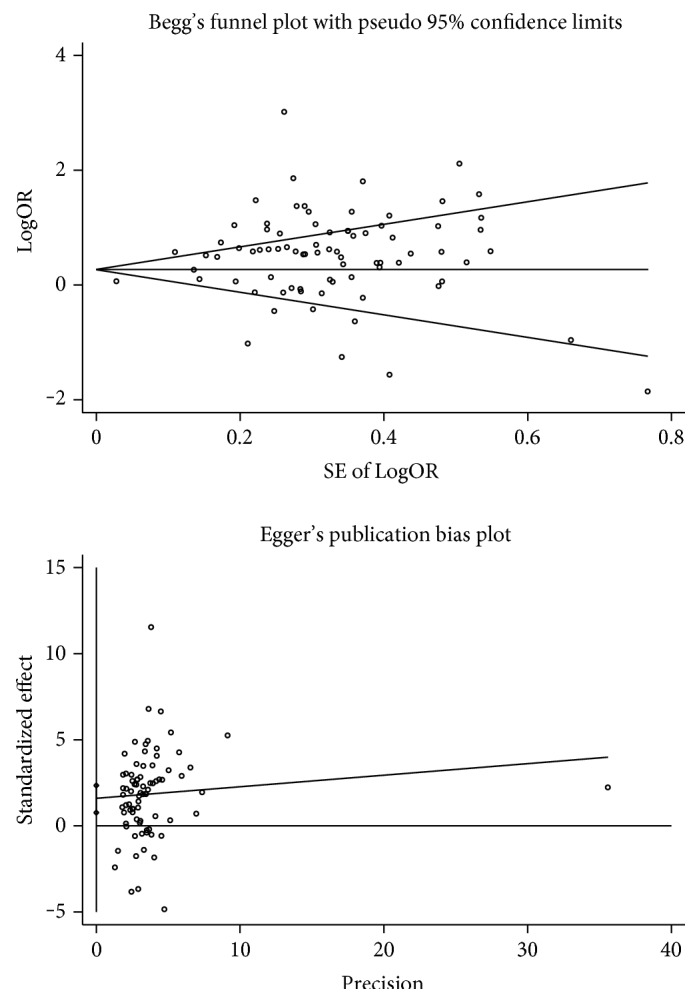
Begg's and Egger's funnel plot of this meta-analysis.

**Figure 8:**
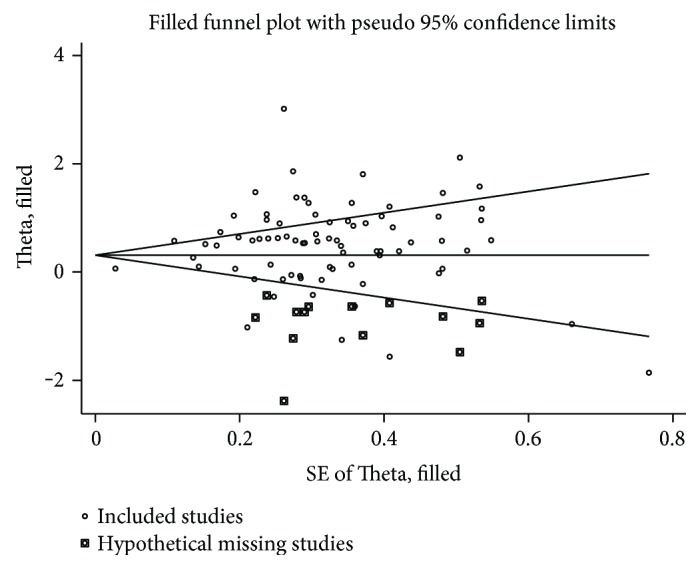
Adjusted funnel plot in the trim and fill method of this meta-analysis.

Funnel plot analysis did not show significant evidences of publication bias (Figure 6). Most of the studies were concentrated symmetrically. No significant publication bias was detected by Begg's test with *Pr* = 0.411 but a significant bias was detected by Egger's test with *P* < 0.001 (Figure 7). As Egger's test indicated the possibility of publication bias, the trim and fill method procedure was performed to identify and correct the publication bias. There was 14 hypothetical missing studies indicated by the trim and fill procedure, and the imputed pooled estimate was 1.366 (95% CI: 1.181–1.580, *P* < 0.001). There still existed a statistically significant association between *H. pylori* infection and DM after adjusting for the publication bias, which suggested that our result was credible. Adjusted funnel plot by the trim and fill method was symmetrical and shown in Figure 8.

## 4. Discussion

DM is a chronic disease characterized by a long-term inflammation mechanism. Guo et al. demonstrated that diabetes was a risk factor for *H. pylori* infection [23]. Several meta-analyses aiming to investigate the association between *H. pylori* infection and DM have been carried out. Zhou et al. recruited 41 studies involving 14,080 patients, and the analysis reported higher risk of *H. pylori* infection among DM patients with OR = 1.33 (95% CI: 1.08–1.64) [12]. Wang et al. retrieved 39 studies involving more than 20,000 participants, with the OR = 1.59 (95% CI: 1.33–1.90) [24]. Our meta-analysis was an updated one and included more studies and individuals. Consistently, we found that the prevalence of *H. pylori* infection was significantly higher in DM patients. But we brought more robust result with higher OR (OR = 1.69, 95% CI: 1.47–1.95; Figure 2). Moreover, we explored more databases and recruited 25 studies reported in Chinese with high-quality score of NOS (all of them were >5). In addition, in subgroup analysis, we found no significant difference in prevalence of *H. pylori* infection in comparison between T1DM patients and non-DM people, which was inconsistent with what was reported by Wang et al. In a subgroup analysis of geographical regions, we found significant higher *H. pylori* infection rate among DM individuals in group Asia and group Europe but not in group Africa or group America. It was inconsistent with the Zhou et al. study which reported that the *H. pylori* effect only happened in Asian people. In this meta-analysis, we found no publication bias with Begg's test, while Egger's test showed a possibility of publication bias. But we performed the trim and fill method and found 14 hypothetical missing studies. The imputed pooled result still supported our original one. Therefore, no publication bias was shown in our meta-analysis and the result we got was credible. In this meta-analysis, the study of Han et al., even though with a total of 30,810 participants, did not affect the significance of the pooled results. Maybe it was because the other studies recruited as enough individuals (a total of 26,587 participants) as to be commensurate to the scale of the Han et al. study. Furthermore, the quality score of NOS for the study Han et al. was 9, which was high. Hence, despite the overweight scale, the study of Han et al. should not be neglected.

We found that there existed an association between *H. pylori* infection and DM in this meta-analysis. Several possible mechanisms might explain the association. Hyperglycemic condition in diabetic individuals could result in immune dysfunction, including damage to the neutrophil function, depression of antioxidant system, and impaired humoral immunity [25]. Moreover, abnormal enteric neuropathy caused by high blood sugar can modulate immune-cell function and stimulate proinflammatory cytokine production, resulting in neurodegeneration [26]. It leads to delay gastric emptying and lacking of acid secretion, which promotes bacterial colonization or overgrowth in gastrointestinal tract [27]. On the other hand, *H. pylori* infection in diabetic patients may worsen glycemic control [28], which leads to the difficulty of DM treatment, forming the vicious circle.

In this meta-analysis, we found that DM patients had a higher prevalence of *H. pylori* infection. But we could not come to the result whether and what role *H. pylori* infection plays on the pathogenesis or development of DM. It was reported that patients could be coinfected with *H. pylori* and some other pathogens like herpes simplex virus 1, cytomegalovirus, and Epstein-Barr virus, some of whom were also associated with DM [29–31]. But the number of researches on this issue was limited. We could not know whether other pathogens affect the effect of *H. pylori* on DM, either. Jeon et al. firstly carried out a prospective cohort study of 782 Latino elderly aged > 60 years and diabetes-free [32]. After following up over 10 years, the authors demonstrated that *H. pylori* seropositive patients experienced a greater rate of incident DM than individuals without DM (hazard ratio 2.69, 95% CI: 1.10–6.60), whereas those who were seropositive for herpes simplex virus 1, varicella virus, cytomegalovirus, and *Toxoplasma. gondii* did not show an increased rate of DM. It indicated that *H. pylori* infection might play an unknown role in the pathogenesis of DM, which implicated a potential step for preventing DM by eradication of *H. pylori* infection. Moreover, it also suggested that other pathogens such as cytomegalovirus and herpes simplex virus 1 might not have the similar effect on the DM like *H. pylori*. But our meta-analysis just revealed the association between *H. pylori* and DM, but could not suggest the effect of *H. pylori* on DM pathogenesis. More researches are needed to find out the actually effect of *H. pylori* infection on DM.

In subgroup analysis based on the types of DM, we demonstrated that 56.5% T2DM individuals were infected with *H. pylori*, but only 36.2% T1DM carried the bacterium (Figure 3). T2DM was more significantly prone to the infection of *H. pylori*. As to T2DM, insulin resistant (IR) is one of its characteristics. Aydemir et al. showed that IR was significantly higher in *H. pylori* infection group [33]. And Eshraghian et al. also supported that *H. pylori* infection was a risk factor for IR [34, 35]. Furthermore, it was reported that IR in T2DM patients could be improved after successful eradication of *H. pylori* [4]. It might partly explain the higher *H. pylori* infection rate in T2DM patients. On the other hand, we found no significant difference in prevalence of *H. pylori* infection in comparison between T1DM patients and non-DM people (*P* = 0.38), consistently with the report by Candelli et al. [27]. Whether this outcome is caused by the different pathogenesis or the onset age of T1DM and T2DM remains unclear. In the T1DM group, the mean age in most studies was not over 20, except for the studies of De Block et al. [36] and Sfarti et al. [37], while in T2DM group, the mean age was usually over 50 years old (Table 1). Epidemiological studies suggested that the prevalence of *H. pylori* infection increases with age [34]. As T1DM mainly onsets during childhood or young age, T1DM patients probably have less chance to be exposed to *H. pylori* infection. Consistently, Krause et al. showed a significantly lower positive rate of antibodies against *H. pylori* in T1DM patients [38]. But some studies held the contrary view that T1DM individuals were also prone to *H. pylori* infection [39, 40]. However, our meta-analysis with pooled estimate favored that T2DM rather than T1DM was associated with *H. pylori* infection. But the sample size of T1DM subgroup was not as large as that of T2DM. Larger sample size is needed to further verify the association between *H. pylori* infection and DM, especially T1DM.

The prevalence of *H. pylori* infection varies in different regions. We found significant higher *H. pylori* infection rate among DM individuals in group Asia and group Europe but not in group Africa or group America (Figure 4). Firstly, it was to be noted that there were much bigger sample size in group Asia and group Europe, respectively. This might be due to the more accurate detection methods and in group Africa and group America; the sample size might be too small to draw robust conclusion. Secondly, it might be explained by that the condition of medical care in developing countries from group Asia was too poor for DM patients to get good control of DM and prevent infectious complications. On the other hand, the epidemiology and different strains of *H. pylori* infection might attribute to the part of the result. Epidemiology studies revealed that almost all the Asians are infected with the strain of *H. pylori* carrying cytotoxin-associated gene A (CagA) but only nearly 60% of western people carried this stain [41, 42]. It was reported that *H. pylori* infection in Asians was predominated by *CagA iceA1* genotypes while Americans and Africans by *CagA iceA2* genotypes [41, 43]. CagA is a major virulence factor of *H. pylori* and has been reported to be associated with diabetic complications [44]. *CagA*-positive strain of *H. pylori* could cause poor glycemic control in T2DM and difficulty in eradication, which might result in the visible *H. pylori* effect among Asian but not African DM patients. However, due to the lack of data, we could not carry out the subgroup analysis based on different strains of *H. pylori*.

A number of testing methods are available for *H. pylori* detection. Serological test, namely, anti-*H. pylori* IgG and/or IgA test, is not affected by acid suppression therapy or recent antibiotic use. But seropositivity could not confirm current *H. pylori* infection, and anti-*H. pylori* IgG titre usually remains elevated for a long period even after clearance or eradication. Some study using anti-*H. pylori* IgG as the diagnosis of *H. pylori* infection might overestimate the infection rate. We typically conducted the analysis of serological test group and current infection group and found that in both subgroups, DM patients had higher prevalence of *H. pylori* infection than non-DM people (Figure 5). As a result, the association between *H. pylori* infection and DM was verified despite of different methods for *H. pylori* detection.

Despite the robust result, there existed limitations in our study. The studies were highly heterogeneous. Variables like age, sex, race, economic status, DM prevalence, and strains of *H. pylori* infection in the included studies varied. For the lack of enough detailed data, subgroup analysis stratified by age, sex, different stages of DM, and strains of *H. pylori*, which might bring up heterogeneity, could not be carried out. Furthermore, most of the articles meeting the inclusive criteria were case-control or cross-sectional ones, and only 3 were prospective ones. More well-designed and prospective cohort studies are needed for clarifying the association between *H. pylori* infection and DM.

In conclusion, despite the limitations, our meta-analysis suggested that there is significantly higher prevalence of *H. pylori* infection in DM when compared with the non-DM individuals. And the difference is associated with type 2 DM but not type 1 DM.

## Figures and Tables

**Table 1:** Characteristics of the included studies.

Author	Year	Country	Study design	Type of DM	Age (years)^♦^	Method of detection^∗^	NOS
Han et al. [20]	2016	China	Cross-sectional	T2DM	64.1 ± 8.6	1	9
Kayar et al. [7]	2015	Turkey	Case-control	T2DM	18–65	2	7
Vafaeimanesh et al. [10]	2015	Iran	Cross-sectional	T2DM	52.84 ± 8.82	3	7
Zhou et al. [14]	2015	China	Case-control	T2DM	42.4 ± 9.8	3, 4	9
Qiao et al. [45]	2015	China	Case-control	T2DM	52.5 ± 1.7	1	7
Ji et al. [46]	2015	China	Case-control	T2DM	51.6 ± 12.5	1, 3	8
Bajaj et al. [9]	2014	India	Case-control	T2DM	≥18	3, 4, 5	8
Chobot et al. [47]	2014	Poland	Case-control	T1DM	13.4 ± 3.4	1	7
Sotuneh et al. [15]	2014	Iran	Cross-sectional	DM	Elderly	3	8
Yang et al. [11]	2014	Taiwan	Cross-sectional	T2DM	59.6 ± 10.0	5	9
Zhang et al. [48]	2014	China	Case-control	DM	52.14 ± 10.25	1	7
Wei et al. [49]	2014	China	Case-control	T2DM	52.79 ± 12.86	1	7
Ye and Xu [50]	2014	China	Case-control	T2DM	54.2 ± 2.0	1	7
Liu et al. [51]	2014	China	Case-control	T2DM	51–65	1	7
Zhou et al. [52]	2014	China	Case-control	T2DM	57.8 ± 11.7	1	7
Wang F and Wang XF [53]	2014	China	Case-control	T2DM	54.6 ± 1.4	1	7
Bai et al. [54]	2014	China	Case-control	T2DM	52.5 ± 14.2	1	7
Jia et al. [55]	2014	China	Case-control	DM	61.0 ± 10.0	1	6
Jafarzadeh et al. [56]	2013	Iran	Cross-sectional	DM	42.86 ± 6.42	3	7
Keramat et al. [57]	2013	Iran	Case-control	DM	51.20 ± 11.60	3, 4, 5	8
Xue et al. [58]	2013	China	Case-control	T2DM	57.03 ± 11.29	1	7
Luo H [59]	2013	China	Case-control	DM	51.5 ± 4.9	4	6
Candelli et al. [60]	2012	Italy	Prospective cohort	T1DM	19.8 ± 4.3	1	7
Jeon et al. [32]	2012	USA	Prospective cohort	DM	67.9 (64.1–71.3)	3	7
Oluyemi et al. [61]	2012	Nigeria	Cross-sectional	T2DM	56.4 ± 10.4	2	7
Hao et al. [62]	2012	China	Case-control	DM	47.24 ± 8.49	1	6
Xu et al. [63]	2012	China	Case-control	T2DM	61.0 ± 10.96	3	7
El-Eshmawy et al. [40]	2011	Egypt	Case-control	T1DM	19.35 ± 2.6	3	7
Wan et al. [64]	2011	China	Case-control	T2DM	53.4 ± 1.8	1	6
Chen et al. [65]	2011	China	Case-control	DM	53.0 ± 5.6	1	6
Agrawal et al. [66]	2010	India	Case-control	T2DM	—	5	7
Devrajani et al. [8]	2010	Pakistan	Case-control	T2DM	>35	2	7
Ibrahim et al. [44]	2010	Egypt	Case-control	T2DM	45 ± 5.4	4, 5, 6	6
Sfarti et al. [37]	2010	Romania	Case-control	T1DM	49.5 ± 14.2	1, 4, 5	7
Xu et al. [67]	2010	China	Case-control	T2DM	51.5 ± 13.0	1	7
Cabral et al. [68]	2009	Brazil	Case-control	T1DM	17.6 ± 1.5	5	6
Ciortescu et al. [69]	2009	Romania	Case-control	DM	—	1, 3, 5	^#^
Krause et al. [38]	2009	Israel	Case-control	T1DM	16.0 ± 8.7	3	6
Lazaraki et al. [70]	2009	Greece	Case-control	T2DM	65.32 ± 8.56	4, 5	6
Zhang LQ and Zhang MQ [71]	2009	China	Case-control	T2DM	56.5 ± 1.1	1	7
Yu [72]	2009	China	Case-control	T2DM	52.5 ± 13.4	1	6
Ariizumi et al. [73]	2008	Japan	Case-control	DM	62.5 ± 11.5	3, 4, 5	6
Demir et al. [74]	2008	Turkey	Case-control	T2DM	52 ± 8.2	5	6
Hamed et al. [75]	2008	Egypt	Case-control	DM	47.65 ± 1.2	3	7
Nicholas et al. [76]	2008	Nigeria	Case-control	T2DM	29–72	3	7
Yan et al. [77]	2008	China	Case-control	T2DM	32–85	1	6
Wang et al. [78]	2008	China	Case-control	T2DM	47.1 ± 6.37	5	6
Ji YF et al. [79]	2008	China	Case-control	T2DM	55.2 ± 13.5	5	7
Bener et al. [80]	2007	Qatar	Case-control	T2DM	48.1 ± 7.9	3	7
Sun et al. [81]	2007	China	Case-control	T2DM	35–85	1	7
Jaber [82]	2006	Saudi Arabia	Case-control	T1DM	Children	3	7
Lu et al. [83]	2006	China	Case-control	T2DM	59.4 ± 11.2	3	7
Gulcelik et al. [84]	2005	Turkey	Case-control	T2DM	51.9 ± 10.6	5	7
Gillum [85]	2004	USA	Cross-sectional	DM	40–74	3	7
Candelli et al. [27]	2003	Italy	Case–control	T2DM	14.8 ± 5.6	1	6
Anastasios et al. [86]	2002	Greece	Cross-sectional	DM	61.4 ± 12.3	5	6
Cenerelli et al. [87]	2002	Italy	Case-control	T2DM	55.7 ± 9.7	1	7
Colombo et al. [88]	2002	Italy	Case-control	T1DM	Children	3	^#^
De Block et al. [36]	2002	Belgium	Case-control	T1DM	41 ± 12	3, 5	7
Maule et al. [89]	2002	Italy	Case-control	T2DM	46–75	1	7
Zelenková et al. [90]	2002	Czech	Case-control	DM	—	3	^#^
Ko et al. [91]	2001	China	Case-control	T2DM	49.9 ± 12.0	4	6
Ivandić et al. [92]	2001	Croatia	Case-control	DM	23–63	5	6
Ravera et al. [93]	2001	Uganda	Case-control	DM	—	5	6
Marrollo et al. [94]	2001	Italy	Case-control	DM	63	5	7
Quatrini et al. [95]	2001	Italy	Case-control	DM	58	1	7
Senturk et al. [39]	2001	Turkey	Case-control	T2DM	—	5, 6	^#^
Vazeou et al. [96]	2001	UK	Case-control	T1DM	14.5	3	6
Xia [97]	2001	Australia	Case-control	DM	60.7 ± 13.3	3	7
Zhao [98]	2001	China	Case-control	T2DM	59.6 ± 1.3	1	6
Arslan et al. [99]	2000	Turkey	Case-control	T1DM	Children	3	^#^
Dore et al. [100]	2000	Italy	Case-control	DM	12–75	3	6
Güvener et al. [101]	1999	Turkey	Case-control	T2DM	—	5	7
Salardi et al. [102]	1999	Italy	Case-control	T1DM	12	3	7
de Luis et al. [103]	1998	Spain	Case-control	DM	24.05 ± 8.3	3	6
Gasbarrini et al. [104]	1998	Italy	Case-control	DM	35 ± 11	1	7
Gentile et al. [105]	1998	Italy	Case-control	T2DM	51 ± 8	5	7
Pocecco et al. [106]	1997	Italy	Case-control	DM	16	4	6
Małlecki et al. [107]	1996	Poland	Case-control	DM	—	5	6

NOS: Newcastle-Ottawa scale. ^♦^Mean age or the range of age in DM group. ^∗^1 = ^13^C or ^14^C urea breath test, 2 = stool antigen test, 3 = anti-*H. pylori* antibody, 4 = rapid urease test, 5 = histology or biopsy, 6 = culture. ^#^Non-English or non-Chinese article or only abstract available which could not get the full text for scoring.

## Abbreviations

*H. pylori*: *Helicobacter pylori*
DM: Diabetes mellitus
T2DM: Type 2 DM
T1DM: Type 1 DM
NOS: Newcastle-Ottawa scale
OR: Odds ratio
CI: Confidence interval
RR: Risk ratio
IR: Insulin resistant
*CagA*: Cytotoxin-associated gene A
MALT: Mucosa-associated lymphoid tissue.
